# Epidemiology of Pediatric Burn Injuries in Isfahan, Iran

**DOI:** 10.5812/atr.5295

**Published:** 2012-06-01

**Authors:** Mohammad Hadi Rafii, Hamid Reza Saberi, Mehrdad Hosseinpour, Esmaeil Fakharian, Mahdi Mohammadzadeh

**Affiliations:** 1 Department of Pediatric Surgery, Isfahan University of Medical Sciences, Isfahan, IR Iran; 2 Trauma Research Center, Kashan University of Medical Sciences, Kashan, IR Iran

**Keywords:** Burns, Adolescents, Pediatrics

## Abstract

**Background:**

Burns are major cause of death and disability worldwide, particularly in the developing countries.

**Objectives:**

The aim of this study was to determine the incidence and causes of burns in children under the age of 15 years in Isfahan province, Iran.

**Patients and Methods:**

All children admitted to the burn center of Isfahan, the largest city in central Iran, between 2007 and 2009 were enrolled in this study. We analyzed the data on age, sex, location, cause and spread of the burn, duration of admission, and cause of mortality.

**Results:**

Out of 2229 burn patients, 1014 (45.5%) were under the age of 15, indicating an annual incidence of 50 in 100,000 children. Of the 1014 patients, 610 (60%) were boys and 404 (40%) were girls; the male-to-female ratio was 1.5:1. Most of the patients were in the age range of 3 to 6 years. Scald was the most common type of burn injury (51.8%). Six-hundred and sixty-eight cases (65.7%) were from urban areas, while 346 (34.3%) were from rural areas. Fifty-six patients (5.5%) died.

**Conclusions:**

Burn injury is a major health concern in the pediatric age group, and specific consideration and planning are required for its management.

## 1. Background

Burns are the third most common cause of mortality in children and adolescents (1). It is also a major cause of morbidity and mortality in individuals of all age groups, particularly in individuals living in the developing countries (2). The epidemiology of burns is diverse across the world and also within a country because of differences in the cultural and socioeconomic factors and the availability of health-care facilities (3). Studies have shown that burns are a major health concern in the Middle East (3); among the countries in the Middle East, Iran has one of the highest populations of children and adolescents, and thus, these populations are at a high risk of burn injuries in Iran (4). In Iran, burns are the second most common cause of death, after traffic accidents, in individuals under the age of 15 years (5). To date, few studies have been conducted on this subject in Iran, and the findings of these studies are rather inconsistent (4,6).

## 2. Objectives

Considering the importance of incidence and causative elements in planning for better control of burns (7), we conducted this study in Isfahan, the largest central city of Iran. 

## 3. Patients and Methods

Isfahan is one of the largest cities in central Iran, with a population of 4,599,256 and having 1,007,881 children under the age of 15 years. All burn patients under the age of 15 years who were admitted to Imam Musa Kazem Hospital of Isfahan University of Medical Sciences, the only burn center in the province, between March 2007 and March 2009 were enrolled in this existing data study. 

The patients were categorized into the following 4 age groups: birth to 24 months, 3–6 years, 7–12 years, and 13–15 years. Sex, cause of burn, place of residence (rural or urban), percentage of spread of burns, duration of hospitalization, and rates of morbidity and mortality were recorded and analyzed using the Chi square test (SPSS-10 software). A *P* value of less than 0.05 was considered statistically significant.

## 4. Results

Of the total 2229 burn patients admitted during the study period, 1014

(45.5%) were under the age of 15 years (annual incidence of burns, 50 cases per 100,000 children). Of these, 610 (60%) were boys and 404 (40%) were girls, and the male-to-female ratio was 1.5:1. In both the sexes, the most common causes of injuries were hot water and steam (51.8%), followed by fire and flame (34.3%). A significantly higher number of boys had burn injuries that did the girls (P = 0.011) (Table 1). 

**Table 1.** Causes of Burns by Sex ^a ^

	Male, No. (%)	Female, No. (%)	Total, No. (%)
Hot vapor and boiling water	291 (55.4)	235 (44.6)	526 (100)
Hot liquid	62 (59.1)	43 (40.9)	105 (100)
Flame	231 (67.3)	114 (32.7)	348 (100)
Electrical	10 (62.5)	6 (37.5)	16 (100)
chemical	13 (68.4)	6 (31.6)	19 (100)
Hot vapor and boiling water	610 (60.2)	404 (39.8)	1014 (100)

^a^*P *= 0.011

Children under the age of 6 years (593) accounted for 58.7% of the patients with burn injuries, making this age group the most vulnerable age group to burns. Hot water and steam were the leading causes of burns, followed by fire, flame, electricity, and chemicals (*P *< 0.001) (*Table 2*). 

**Table 2.** Causes of Burns by Age Group ^a ^

	0-2, y, No. (%)	3-6, y, No. (%)	7-12, y, No. (%)	13-15, y, No. (%)	Total, No. (%)
Hot vapor and boiling water	213 (40.4)	207 (39.3)	84 (15.9)	22 (4.4)	526 (100)
Hot liquid	65 (61.9)	26 (24.7)	10 (9.5)	44 (3.9)	105 (100)
Flame	12 (3.6)	59 (16.9)	130 (37.3)	147 (42.2)	348 (100)
Electrical and chemical	1 (2.8)	10 (28.5)	17 (48.5)	7 (20.2)	35 (100)
Total	291 (29)	302 (29.7)	241 (23.7)	180 (17.6)	1014 (100)

^a^*P *< 0.001

Six hundred and sixty-eight (65.7%) cases were from urban areas, while 346 (34.3%) were from rural areas. In urban regions, the most common causes of burn injuries were hot water and steam (368 cases (55.1%)), followed by fire and flame (205 cases (30.7%)), hot liquids (73 cases (10.9%)), electricity (12 cases (1.8%)), and other causes (10 (1.5%) cases). In rural areas, the most common causes of burn injuries were hot water and steam (158 cases (45.7%)), fire and flame (143 cases (41.3%)), hot liquids (32 cases (9.2%)), electricity (4cases (1.2%)), and other causes (9 cases (2.6%)).

Spread of burn of more than 40% is more common in case of burn injuries caused by fire and flame. In 39% of the general burn injuries, an 11–20% spread of burns was observed *(Table 3)*.

**Table 3.** Causes of Burns by TBSA ^a^^, b^

	10 ≥, No. (%)	11-20, No. (%)	21-40, No. (%)	41 ≤ , No. (%)	Total, No. (%)
Hot vapor and boiling water	138 (26.2)	239 (45.4)	129 (24.5)	20 (3.9)	526 (100)
Hot liquid	45 (42.9)	42 (40)	16 (15.3)	2 (1.8)	105 (100)
Flame	66 (18.9)	111 (31.8)	107 (30.7)	62 (18.6)	348 (100)
Electrical and chemical	27 (77.2)	4 (11.4)	2 (5.7)	2 (5.7)	35 (100)
Total	276 (27.2)	396 (39)	254 (25)	88 (8.8)	1014 (100)

^a^*P* < 0.001

^b^Abbreviation: TBSA,Total body surface area

The range for length of hospital stay was 1–62 days (7.5 ± 3.2). Three hundred and ninety-one (38.5%) patients were admitted for less than 3 days; 297 (29.2%), 4–10 days; 281 (27.7%), 11–30 days; and 45 (4.4%), more than 30 days. 

Fifty-six (5.6%) patients died. Of these, 38 (67.8%), 15 (26.8%), and 2 (3.6%) patients died because of injuries caused by fire and flame, hot water and steam, and hot liquids, respectively. One (1.8%) patient died because of other injuries. 

## 5. Discussion 

The annual incidence of burns in children aged less than 15 years in Isfahan province is 50 per 100,000 children. This incidence is higher than that in the other parts of the country, including that in Tehran (annual incidence, 20.8 per 100,000 children) (6) and Hamedan (33.4 per 100,000 children) (8), and in Kuwait (17.5 per 100,000 children) (9) and Hong Kong (23 per 100,000 children) (10). Few studies (11, 12) have reported incidences higher than that reported in this study. The high incidence in our study may be attributable to the number of referrals from neighborhood provinces, including Chaharmahal and Yazd. Specific socio-economic and cultural factors may also contribute to this high incidence rate, and they need to be further investigated.

In this study, 45.5% of the patients admitted to the burn center were children aged less than 15 years. This finding is similar to those reported in other studies from Iran (6), Egypt (7), and Turkey (13). The incidence is higher than that reported in Hamadan (29.3%) (8) and Turkey (35%) (7). The reason for this high incidence may be attributable to better attention to childhood problems, which results in the referral of these patient groups to specialized treatment centers. However, this high incidence may also be an important indicator of a higher risk of burns in Iranian children, especially when the incidence rate has remained steady across different studies (6). An important point is that the reported incidence is only of hospitalized patients; and hence, the actual incidence of the general population may be different.

The mortality rate in our study was 5.5%, which is lower than those found in previous studies in Iran (6, 8), and is similar to those reported in Turkey (7) and China (14); however, it is higher than that reported in the other countries (9, 15). Although it appears that the incidence of burns in Iran is largely unchanged over time, the mortality rates seem to have dropped. The drop in mortality rates may be attributable to better diagnosis, management, and referral to specialized centers. In spite of the differences in age classification in different studies, in most of the studies, children under the age of 6, and particularly under the age of 3, form the most vulnerable group, similar to our study (3, 6, 13, 14, 16-21). In our study, the male-to-female ratio was 1.5:1. A similar finding was observed in some other studies (21, 22); however, certain other studies showed that burn injuries were more common in adolescent girls who were exposed to cooking activities (7, 22). In our study, like many other studies, scalding was the most common type of burn injury, particularly between the ages of 3 and 6 years (15, 23, 24). In France and Iceland, hot liquids are the leading causes of burn injuries (18, 25). This finding may be attributable to the very common use of kettles and samovars for preparation of tea, as in our country. The number of burn injuries caused by fire and electricity increase in children after the age of 6, owing to an increase in their activity and curiosity levels; the increased incidence in children aged more than 6 years in developing countries may also be attributable to the children’s involvement in professional activities (6, 13). In this study, the incidence of burns reported in the urban regions was higher than that in the rural areas; however, burns caused by flame and fire were more common in the rural areas, probably owing to the use of fire for baking and warming in households. The higher percentage of burns and mortality in this kind of injury suggests a need for further investigation for the prevention of this problem. The locations of burns on the body in our study are similar to those reported in other studies (26, 27), which may be attributable to the similarity of the causative factors. This study showed that although the rate of mortality due to burn injuries has decreased in Iranian children under the age of 15 years, the incidence of burn injuries remains high in this population. This finding necessitates planning for better prevention of burn injuries by maintaining higher manufacturing standards for household utensils and educating the public about burn injuries.
